# Development and Validation of a Social Media Questionnaire for Nursing Training: A Pilot Study

**DOI:** 10.3390/healthcare9030344

**Published:** 2021-03-18

**Authors:** Diana Jiménez-Rodríguez, María Teresa Belmonte García, Jesús Arcos García, Gracia Castro-Luna

**Affiliations:** 1 Department of Nursing, Physiotherapy and Medicine, University of Almeria, 04120 Almeria, Spain; d.jimenez@ual.es (D.J.-R.); graciacl@ual.es (G.C.-L.); 2 Surgical Service, Rafael Mendez Hospital, 30813 Lorca, Spain; jag850@inlumine.ual.es

**Keywords:** undergraduate nursing students, social media, nursing education, tool validation

## Abstract

Background: Social media platforms are integrated into the lives of students. Their use in education has been studied, but this research is scarce in nursing. The objective of this study was to develop and validate the questionnaire “Use and views of the social media for nursing education” through a pilot study, to describe the use and attitudes of nursing students to social media. Methods: Cross-sectional design to validate the modified scale “Students’ Use and Views of the Social Media questionnaire.” The sample consisted of 107 undergraduate nursing students. Results: The factor analysis extracted three main components to explain social media use for nursing education, with component 1 being the “Need to use media in my professional training,” component 2—“To deepen my professional knowledge” and component 3 “Contrast information.” High reliability was demonstrated with Chronbach’s alpha value (0.84). Conclusion: The final tool was proven to have high validity and reliability values, so it is positioned as a viable tool to explore this reality. Students use social media for education in a high proportion and have positive attitudes regarding their education inclusion.

## 1. Introduction

Social media can be defined as recognizable websites and mobile applications that give the opportunity to communicate, collaborate, connect with others and share opinions and experiences through images, videos or audio clips. Social media promote participation by allowing continuous dialog and feedback [1]. 

Social media are integrated into the lives of young people, including university students. They report that they most often use them to watch movies, interact with friends and play games [2].

As mentioned in the literature, the use of social media for health sciences students’ training has been researched. However, this research is scarce and much more so when we focus on nursing students [1,2,3,4,5]. It may be partly due to the time it took the nursing discipline to integrate social media into training [6,7], as early studies on this subject reflected that nursing students indicated their preference for learning following the traditional teaching method [3]. Although social media are used in higher proportion for personal relationships, there is evidence of their use for nursing students’ educational purposes [1,2,3]; the latest research on social media learning places health sciences with other disciplines such as applied sciences, management, information science, psychology and social sciences as the areas especially interested in this subject [8]. 

The studies reviewed present different results in terms of the use of social media for training. For instance, the first published articles showed that students preferred receiving information following the traditional teaching method [3]. However, the latest published studies observed a positive effect of social media on training [2,4]. 

An important point that we could not ignore was to consider the attitudes and implications on the part of educators and institutions in the use of social media to train students. A clear line of debate is introduced regarding this fact, which appears scarcely reflected in the literature in the case of nursing students [9]. Recently, Pizzuti et al. [10] identified perceptions of using social media as an educational tool among healthcare practitioners. In this study, healthcare practitioners frequently utilized social media, and many believe it can be a useful educational tool in healthcare. Another research showed students’ positive perception of the use of social media in education. For instance, they stated that they improved communication dynamics with teachers, sensed that they were learning more throughout the process [11]. In short, the importance of social media in higher education students is undeniable. However, many teachers believe that social media in classrooms hinder learning and can be disruptive because they can be a source of distraction [12].

Social media is effective for the training of nursing students [2,3,13]. Still, the literature concludes that a positive attitude towards using social media for training is necessary to incorporate them efficiently into the educational environment [2,14]. Therefore, it is essential to know the attitudes of students towards them. 

Due to the importance and impact that social media currently have on students’ lives, it is essential to describe and know their attitudes and the use they make of them for their professional training. Thus, this study’s objective was to develop and validate the modified scale “Students’ Use and Views of the Social Media questionnaire” as well as to understand the use of social media for training of nursing students.

## 2. Materials and Methods

### 2.1. Design

A cross-sectional study. The main objective consisted in developing and validating the modified scale “Students’ Use and Views of the Social Media questionnaire” [2]. First, the scale’s adaptation was carried out; in the second place, the scale was modified with the inclusion of the items intended to measure the use of social media for training in nursing. Finally, the questionnaire was validated, and a descriptive pilot study was carried out.

### 2.2. Sample and Participants

A convenience sample was obtained among undergraduate nursing students from the University of Almeria (UAL), Spain. The sample was recruited between April and May 2019 among the nursing students who regularly attended theoretical and practical classes. Furthermore, participation was voluntary, and students did not receive any gratification or reward for their participation in the study. The students who agreed to participate in the study were asked to complete an online questionnaire. Most students in the sample were in the second (41.6%) or third (52.9%) undergraduate nursing year. The sample of the study consisted of 107 students. The sample size estimation was based on Prof. Hair’s recommendations of at least 100 individuals with at least five individuals per variable (12 items) analyzed [15].

### 2.3. Instrument

The “Students’ Use and Views of the Social Media” questionnaire initially developed by Maurice Hall in his study of pharmacy students in the UK [16] (Appendix A) and subsequently validated for nursing students in the Arabic culture by Noah Al-Shdayfat [2] was used as reference. Both authors were contacted via e-mail to gain access to the original instrument and authorization for its use. Maurice Hall’s questionnaire consisted of questions and statements to consider about social networking divided into four sections: use of social networking (6 items), online privacy and profile (5 items), professionalism and social networking (8 items) and Student demographics (2 items). 

### 2.4. Procedure

First, a direct and inverse translation of the original scale was made. A focus group of six nurses aged 25–40 years discussed whether a sworn translator’s first translation suited the subject matter. The content of the questionnaire was accepted as valid. It was decided to maintain the translation, and then an inverse translation was performed, which was again evaluated by the focus group. Finally, the translation was reviewed by a group of experts who approved it.

After the analysis of the questionnaire, modifications were made to adapt it to the Spanish culture and our research, which aimed to understand the use of social media for specific nursing training (Appendix A).

Finally, a self-administered questionnaire was developed that consisted of three sections that sought to reflect the use students make of social media for their academic training:

- Demographic data;
- Generalities of the use of social media (12 multiple-choice questions that aim to describe the students’ use of social media);
- Specific use for training, divided in turn into:
  - Use of social media information (eight items in Likert format from 1 to 5 (1 = totally disagree, 5 = totally agree), which are intended to describe the specific use for training);
  - Attitudes towards the use in learning (five questions in Likert format from 1 to 5, which aim to explore students’ attitudes and expectations regarding the use of social media in their training).
- The participants were provided with a link to the questionnaire using application Google Forms™ (Alphabet, Mountain View, CA, USA). No time limits were set to complete it. Before completing the questionnaire, the students were informed of the study design and objectives.

### 2.5. Data Analysis

Data analysis was performed using IBM SPSS v.25 and R v.3.5.1. Quantitative variables were analyzed using central trend measures and dispersion measures (standard deviation). Qualitative variables were analyzed in terms of percentages and frequencies. As for exploratory factorial analysis, the main component extraction method was carried out to evaluate the construct’s validity. The skewness and the kurtosis were explored for all items of the questionnaire. The tool’s reliability was measured in terms of internal consistency using Chronbach’s alpha analysis and homogeneity using correlation coefficients.

### 2.6. Ethical Aspects

The approval for this research was obtained by the Ethics and Research Commission of the Department of Nursing, Physiotherapy and Medicine of the University of Almeria (No. EFM-27/19). The questionnaire was anonymous. All the participants consented to participate in the study. The study was carried out following the ethical aspects of the Declaration of Helsinki [17].

## 3. Results

### 3.1. Characteristics of the Sample

The sample consisted of a total of 107 participants. The age of the participants ranged from 18 to 35 years, with the average age of 21.69 ± 3.11. We found a predominance of women with 78.5% as compared to men (21.5%; *n* = 23). On the other hand, regarding the course, 57% (*n* = 61) of the participants were in the third year of the nursing degree, 35.5% (*n* = 38) were second-year students, 4.7% (*n* = 5) were fourth-year students and 2.8% (*n* = 3) were first-year students.

### 3.2. Generalities of the Use of Social Media 

Social media use was reported by 100% (*n* = 107) of the sample. The most commonly used social medium was Instagram (*n* = 102), followed by YouTube (*n* = 91), Facebook (*n* = 81), Twitter (*n* = 41) and Pinterest (*n* = 16). 

As for the main reason for the use of social media, for 100 participants (93.5%), it was personal use, 36 participants (33.6%) selected education and 22 (20.6%)—professional use. As for the time spent using social media, 41.1% (*n* = 44) used them for 2–3 h, 23.4% (*n* = 25)—formore than 3 h, 22.4%—between 1–2 h, 12.1% (*n* = 13)—for30 min to 1 h and 0.9% (*n* = 1)—forless than half an hour. 

A vast majority of the participants (96.3%; *n* = 103) reported using social media to increase career knowledge, prepare for an exam or ask questions. The social medium most used for this purpose was YouTube (*n* = 77), followed by Instagram (*n* = 64) and Facebook (*n* = 31). 

A majority of the participants (89.7%; *n* = 6) reported following an account dedicated to sharing educational content on nursing. Instagram was the social medium with the most institutional accounts followed (*n* = 89), followed by Facebook (*n* = 39) and YouTube (*n* = 18). These profiles, according to the respondents, were managed by nursing professionals (*n* = 93), doctors (*n* = 46), institutions (*n* = 11), educational disseminators (*n* = 11), teachers (*n* = 8) or unqualified personnel (*n* = 4).

Most sample participants (77.2%; *n* = 80) stated that they consumed educational content by chance with the rest of the content on social media, compared with 50.05% (*n* = 53) who expressly indicated searching for it.

As for how they learned about the social media’s educational use, 75 participants learned about it through classmates, 32—through family and friends, 20—through professionals and 20—through teachers. Most participants (74.8%; *n* = 80) said they had recommended using social media for learning to other students.

### 3.3. Scale Validation and Factorial Analysis

Table 1 shows the descriptive statistics for all items of the questionnaire. The skewness and the kurtosis evaluate the shape of distribution for each item of the questionnaire. The standard error of skewness and kurtosis are to obtain the test statistic, which measures how many standard errors separate the sample skewness and kurtosis from zero; if the standard error is between −2 and +2, it may be symmetric. 

An exploratory factorial analysis was carried out to verify the construction validity of the tool developed. Kaiser–Meyer–Olkin test (KMO) had a result of 0.849. There was significance when performing Bartlett’s sphericity test (X2 (78) = 455.819, *p* < 0.05). Both tests demonstrated the appropriateness of performing a factorial analysis. A correlation matrix was built to assess how much each item was saturated before the exploratory factor analysis by analysis of the main components and varimax rotation. Self-values above 1 identified three components with which 58.39% of the variance in questionnaire scores was explained.

The rotated component analysis was carried out for the three factors obtained after the factorial analysis. The rotated component matrix specifying in which component each item is located is reflected in Table 2. The components were defined as follows:

1. Component 1 “Need to use media in my professional training” was composed of six items, explained 36.74% of the variance.
2. Component 2 “To deepen my professional formation knowledge” was composed of four items, explained 13.28% of the variance.
3. Component 3 “Contrast information” was composed of two items, explained 8.35% of the variance.

### 3.4. Reliability of the Instrument

Cronbach’s alpha test was carried out to determine the internal consistency of the tool developed (α = 0.84) and demonstrated high reliability. All the items displayed an item-to-total correlation > 0.30 (Table 3).

### 3.5. Mean Score of Each Item and the Frequency of Endorsement of the Final Questionnaire

Table 4 reflects each item’s mean values and the standard deviation and the frequency of endorsement for each response. The students expressed high mean values on a Likert scale for 10 of the 12 items that made up this part of the questionnaire. However, endorsement frequencies were very low for values 1, 2 and 3 of the scale (less than the desirable 20%), possibly due to a problem of “social desirability.” 

### 3.6. Evaluating the Model Fit. Confirmatory Factor Analysis

Table 5 shows the confirmatory factor analysis indices. Chi-squared statistics compare the fit of nested models to the data. It is not helpful if the data size is small. The RMSEA (root mean square error of approximation) ranges from 0 to 1, with smaller values indicating better model fit. The standardized root mean square residual (SRMR) ranges from 0 to 1, with a value of 0.08 or less indicating an acceptable model. The GFI (goodness of fit index), AGFI (adjusted goodness of fit index), NFI (normed fit index), non-normed fit index (NNFI; also known as the Tucker–Lewis index) and CFI (comparative fit index) range between 0 and 1 and showed a good model fit. 

## 4. Discussion

The study’s objectives consisted of the evaluation with general questions of the use and attitudes to social media for training by nursing students and the pilot study for the development and validation of the questionnaire “Use and views of the social media for nursing training”.

Regarding social media as a tool for education, our results showed that 33.6% of the students referred to education as the main reason for social media use, while 96.3% reported having used them for this reason. Regarding education as the primary use, our results are consistent with various studies in this field with an approximate percentage of 20–30% [1,2,3]. Logically, the first use is personal since social networks have become part of our lives. The proportion of participants who claimed to have used social media for educational purposes at one time in our research is striking (96.3%), as it is significantly higher than in other studies (60–80%) [2,13]. However, this may be due to uncontrolled factors such as culture or student motivation. These data suggest using social media as a tool for reinforcing knowledge rather than for education itself. 

The bibliography consulted coincides in pointing to Facebook and YouTube as the social media most used by students [3,5]. Although our sample used both social media, Instagram is the social site most used by our participants. In this sense, the existing publications point to the increased use of Instagram in general [13,14]. Our results maintain the trend of the latest publications [14]. Instagram is positioned as the most widely used social network by nursing students.

Concerning our second objective, the questionnaire’s validation was carried out with factorial analysis using the method of analysis of the main components with varimax rotation. The factorial analysis extracted three components that explained 57% of the variance as compared to the original questionnaire validated by Al-Shdayfat [2] that had only one factor capable of explaining 74%. It may be due to the modifications made to the items added to respond to the research objective and sample size differences.

The reliability study showed a Chrombach’s alpha equal to 0.84, which matches the data also obtained by Al-Shdayfat [2] to validate the questionnaire for students in the Arabic culture. 

Although studies have measured the use of social media through other scales [2,13], these have focused on the effect of social media on students’ professional careers, taking into account facts as if they are aware of privacy terms when dealing with patients. Our scale’s modifications are intended to describe social media use specifically for training, i.e., how nursing students use social media to implement knowledge in their learning. 

From the evaluation of the items in our questionnaire, it can be deduced that our students ensured that they consumed educational content and the rest of the social media content. This content was also consumed through profiles controlled mainly by healthcare personnel; this is not reflected in the literature, which constitutes a new research route. This fact is related to the students’ capacity to use new technologies and adapt them for their training [14,18]. It is frequently mentioned in the literature that students positively perceive the use of social media for training. Studies with a qualitative focus could be carried out to investigate students’ attitudes [19].

The bibliography coincides with the positive use of social media to achieve educational objectives [1,3,13,14]; however, the implementation of strategies is necessary [3,13] since most nursing schools do not even have specific guides or training programs for them [20]. The participants expressed that they would like to use social media in the future; however, as D’Souza et al. [9] indicate, one of the difficulties lies in the lack of knowledge among educators regarding the way to integrate social media into the classroom. 

Finally, we agree with Terkes et al. [14] on the need to continue research in this field to carry out an in-depth study on the attitudes and perceptions of students regarding the use of social media in different contexts and cultures, since this could guarantee efficient implementation of social media in the training of nursing students. Furthermore, social media have an essential utility in education and more so in these times of the pandemic. Therefore, we believe it is necessary to continue researching in this field and check if the results regarding the use or opinions on social media will change after the pandemic in addition to exploring other social networks that have increased in use, all this to improve teaching through the use of all available resources.

### Study Limitations

Among the limitations of this study is the use of a convenience sample limited to a university and early vocational training. Furthermore, the sample size is small, which does not allow the population’s data representativeness.

Although the instrument developed is highly reliable (α = 0.84), factor analysis showed that the use of three components explains only 57% of the variance. Studies with a broader scope should be carried out to determine the results’ stability in larger samples. Finally, instant messengers such as WhatsApp were not included in the study since these social media platforms limit the content and dissemination of information.

## 5. Conclusions

The development of the scale “Use and views of the social media for nursing training” resulted in a scale of 12 items divided into three domains. The final tool was proven to have high validity and reliability, so it is positioned as a viable tool to explore this reality. 

A high proportion of students use social media for education. This educational use usually takes place in a relaxed environment together with other social media content. This fact constitutes a new way of perceiving the use of social media for training.

The students present positive attitudes towards its use, although mostly to reinforce knowledge or acquire knowledge that they did not receive via their educational programs. We believe that a more in-depth study is needed to apply social media in the classroom to achieve specific educational objectives.

## Figures and Tables

**Table 1:** Descriptive statistics.

Questionnaire Items	*n*	Mean	Std. Deviation	Skewness		Kurtosis	
	Statistic	Statistic	Statistic	Statistic	Std. Error	Statistic	Std. Error
Item 1.1	107	3.71	0.89	−0.37	0.23	−0.13	0.46
Item 1.2	107	3.43	1.08	−0.52	0.23	−0.20	0.46
Item 1.3	107	3.33	1.08	−0.32	0.23	−0.34	0.46
Item 1.4	107	3.88	0.94	−0.73	0.23	0.41	0.46
Item 1.5	107	3.84	1.03	−0.89	0.23	0.52	0.46
Item 1.6	107	3.31	1.18	−0.45	0.23	−0.66	0.46
Item 1.7	107	3.55	0.79	−0.40	0.23	0.31	0.46
Item 1.8	106	3.42	1.10	−0.45	0.23	−0.34	0.47
Item 2.1	107	3.93	0.85	−0.44	0.23	−0.41	0.46
Item 2.2	107	3.44	1.01	−0.47	0.23	−0.06	0.46
Item 2.3	107	4.08	0.87	−1.04	0.23	1.61	0.46
Item 2.4	107	3.82	0.97	−0.58	0.23	0.27	0.46
Item 2.5	107	4.21	0.82	−0.92	0.23	0.98	0.46

**Table 2:** Rotated component matrix.

Questionnaire Items	Component		
	1	2	3
2.4. I would like to use social media to consolidate knowledge or review the material learned in class	0.805	0.295	
2.5. I would like teachers to recommend quality content from social media	0.798		
2.3. I consider the use of social media useful for learning	0.764	0.348	0.204
2.2. I assimilate new knowledge better in the context of learning through social media than in the master class context	0.705	0.143	
2.1. I feel that the use of social media is useful for learning the profession of nurse	0.551	0.424	0.235
1.8. I have used the knowledge I obtained from the social media in clinical practice	0.458	0.380	0.217
1.1. I have applied the knowledge I obtained on the social media in some of the areas of the career		0.805	
1.3. Learning through social media will help me to find out better answers for the exam	0.289	0.764	
1.5. I have used the social media to reinforce some aspect of the syllabus of a subject	0.209	0.513	0.495
1.2 The use of social media motivated me to study the subject more deeply	0.295	0.505	0.324
1.4 I have learned new knowledge related to nursing which I had not studied in the different areas of the career.	0.327	0.392	0.170
1.6 I usually check the information I find out on social media about nursing			0.873
1.7. The information I get from the social media is in line with the knowledge I have	0.134	0.201	0.596

**Table 3:** Reliability for each component of the scale.

Means Components	Cronbach’s Alpha	*n*
Component 1	0.83	6
Component 2	0.72	5
Component 3	0.36	2

**Table 4:** Average score per item and frequency of endorsement.

Item	Mean ± SD	1 *n* (%)	2 *n* (%)	3 *n* (%)	4 *n* (%)	5 *n* (%)
1.8. I have used the knowledge I obtained from the social media in clinical practice	3.41 ± 1.10	7 (6.5%)	13 (12.1%)	32 (29.9%)	37 (34.6%)	17 (15.9%)
2.1. I feel that the use of social media is useful for learning the profession of nurse	3.93 ± 0.84	0	6 (5.6%)	24 (22.4%)	48 (44.9%)	29 (27.1%)
2.2. I assimilate new knowledge better in the context of learning through social media than in the master class context	3.43 ± 1.01	5 (4.7%)	12 (11.2%)	35 (32.7%)	41 (38.3%)	14 (13.1%)
2.3 I consider the use of social media useful for learning	4.08 ± 0.87	2 (1.9%)	3 (2.8%)	18 (16.8%)	48 (44.9%)	37 (34.6%)
2.4 I would like to use social media to consolidate knowledge or review the material learned in class	3.82 ± 0.96	3 (2.8%)	3 (2.8%)	34 (31.8%)	37 (34.6%)	30 (28%)
2.5. I would like teachers to recommend quality content from social media	4.20 ± 0.82	1 (0.9%)	1 (0.9%)	18 (16.8%)	42 (39.3%)	45 (42.1%)
1.1. I have applied the knowledge I obtained on the social media in some of the areas of the career	3.71± 0.89	1 (0.9%)	8 (7.5%)	32 (29.9%)	46 (43%)	20 (18.7%)
1.2. The use of social media motivated me to study the subject more deeply	3.42 ± 1.08	7 (6.5%)	12 (11.2%)	32 (29.9%)	40 (37.4%)	16 (15%)
1.3. Learning through social media will help me to find out better answers for the exam	3.32 ± 1.07	7 (6.5%)	14 (13.1%)	38 (35.5%)	33 (30.8%)	15 (14%)
1.5. I have used the social media to reinforce some aspect of the syllabus of a subject	3.84 ± 1.02	4 (3.7%)	7 (6.5%)	21 (19.6%)	45 (42.1%)	30 (28%)
1.6. I usually check the information I find out on social media about nursing	3.30 ± 1.17	10 (9.3%)	17 (15.9%)	5 (23.4%)	40 (37.4%)	15 (14%)
1.7. The information I get from the social media is in line with the knowledge I have	3.55 ± 0.79	1 (0.9%)	8 (7.5%)	38 (35.5%)	51 (47.7%)	9 (8.4%)

**Table 5:** Confirmatory Factor Analysis.

Confirmatory Index	Value
Model chi-squared *p*	0.03
Goodness of fit index	0.89
Adjusted goodness of fit index	0.84
RMSEA index	0.06
Bentler–Bonett NFI	0.82
Tucker–Lewis NNFI	0.93
Bentler CFI	0.94
SRMR	0.07

## Data Availability

The data presented in this study are available on request from the corresponding author.

## Appendix A. Modified Questionnaire Used in the Present Study

### Appendix A.1. Data Demographics

- Age
- Sex
- Academic course

### Appendix A.2. Generalities of the Use of Social Media

Do you use social media?

- [ ] YES
- [ ] NO

What type of social media do you use? Tick the relevant box.

- [ ] Facebook
- [ ] Twitter
- [ ] Instagram
- [ ] Pinterest
- [ ] Youtube
- [ ] Other. Please specify

The main reason why you use social media:

- [ ] Education
- [ ] Personal
- [ ] Professional

On a typical day, how much time do you spend cheking your social media?

- [ ] Less than half an hour
- [ ] 30 min–1 h
- [ ] 1–2 h
- [ ] 2–3 h
- [ ] More than 3 h

Have you ever used the social media to increase your knowledge about your degree/to prepare for an exam/to check answers to questions? It is reformulated due to the original instrument of Likert scale.

- [ ] YES
- [ ] NO

If this is the case, which one?

- [ ] Facebook
- [ ] Twitter
- [ ] Instagram
- [ ] Pinterest
- [ ] Youtube
- [ ] Other. Please specify

Do you follow an account dedicated to sharing educational content about nursing?

- [ ] YES
- [ ] NO

If this is the case, in which social media?

- [ ] Facebook
- [ ] Twitter
- [ ] Instagram
- [ ] Pinterest
- [ ] Youtube
- [ ] Other. Please specify

Who manages this profile?

- [ ] Nursing professional
- [ ] Doctor
- [ ] Institution
- [ ] Educational disseminator
- [ ] Faculty
- [ ] Unqualified personnel
- [ ] Other. Please specify

How do you access this type of educational content on social media?

- [ ] Casually, with the rest of the content that I follow on social media.
- [ ] I look for it specifically
- [ ] Other. Please specify

How did you find out about this educational use of social media?

- [ ] Family and friends
- [ ] Classmates
- [ ] Professionals
- [ ] Teachers
- [ ] Other. Please specify

Have you ever recommended the use of social media for learning to other students?

- [ ] YES
- [ ] NO

### Appendix A.3. Likert (Specific Use and Attitudes)

Next, you will answer questions following a Likert scale where:

1 means ”Totally disagree”

5 means ”Totally agree”

#### Appendix A.3.1. Use

The following questions are intended to measure the impact that the use of social media may have had on your training. If you have never used social media for this purpose (never seen an explanatory video on Youtube or any other social media), please leave the section blank and continue with question 9.

1. I have applied the knowledge I obtained on the social media in some of the areas of the career.
2. The use of social media motivated me to study the subject more deeply.
3. Learning through social media will help me to find out better answers for the exam.
4. I have learned new knowledge related to nursing which I had not studied in the different areas of the career.
5. I have used the social media to reinforce some aspect of the syllabus of a subject.
6. I usually check the information I find out on social media about nursing.
7. The information I get from the social media is in line with the knowledge I have.
8. I have used the knowledge I obtained from the social media in clinical practice.

#### Appendix A.3.2. Attitudes

9. I feel that the use of social media is useful for learning the profession of nurse.
10. I assimilate new knowledge better in the context of learning through social media than in the master class context.
11. I consider the use of social media useful for learning.
12. In the future, I would like to use social media to consolidate knowledge or review the material learned in class.
13. In the future, I would like teachers to recommend quality content from social media.
